# IS*Aba1‐*mediated *crp‐osmC* amplification enhances host‐associated fitness in *Acinetobacter baumannii*

**DOI:** 10.1002/mlf2.70105

**Published:** 2026-09-14

**Authors:** Da‐Wei Wei, Jiangqing Huang, Hailing Fang, Xinyu Wang, Gang Zhang, Chao Wang, Yuqin Song, Jie Feng

**Affiliations:** ^1^ State Key Laboratory of Microbial Diversity and Innovative Utilization, Institute of Microbiology Chinese Academy of Sciences Beijing China; ^2^ College of Life Science University of Chinese Academy of Sciences Beijing China

**Keywords:** *Acinetobacter baumannii*, *crp‐osmC* amplification, host‐associated fitness, IS*Aba1*, ST2 lineage

## Abstract

*Acinetobacter baumannii* is a pathogen, highly adaptable to both hospital‐ and host‐associated environments. Understanding the genetic mechanisms underlying its adaptability is critical for controlling its persistence. Our study identified the *crp‐osmC* gene cluster, flanked by IS*Aba1* elements, as a significant genetic structure undergoing amplification in *A. baumannii*. By experimentally mimicking the amplification of this gene cluster, we found that it substantially enhanced bacterial growth and competitive fitness, promoted survival in mouse serum, and improved colonization in murine infection models. Mechanistically, transcriptomic analysis suggested that *crp‐osmC* amplification may enhance host‐associated fitness by promoting pathways related to metabolic flexibility and nutrient acquisition, including phenylalanine metabolism, siderophore biosynthesis, and sulfur metabolism. It is noteworthy that this amplification was largely limited to the epidemic ST2 lineage clinical isolates. Intriguingly, gene amplification was also accompanied by reduced biofilm formation and heightened susceptibility to several antibiotics. Overall, IS*Aba1*‐mediated amplification of the *crp‐osmC* cluster enhances host‐associated fitness in *A. baumannii*, while the accompanying increase in antibiotic sensitivity reflects a trade‐off between survival and resistance.

## Impact statement

The clinical success of *Acinetobacter baumannii* reflects its notable adaptability and persistence. This study identifies IS*Aba1*‐mediated amplification of the *crp‐osmC* locus as a previously unrecognized mechanism that enhances host‐associated fitness in the globally prevalent ST2 lineage by promoting bacterial growth competitiveness, serum resistance, and host colonization. Notably, this amplification also reduced biofilm formation and increased antibiotic sensitivity, revealing a trade‐off between host adaptation and resistance. These findings uncover a link between insertion sequence‐driven gene amplification and host adaptation, providing insights into the genetic basis of *A. baumannii* evolution and its clinical predominance.

## INTRODUCTION

Within just a few decades, *Acinetobacter baumannii* has rapidly adapted to clinical settings, becoming a leading nosocomial pathogen worldwide^1^, ^2^, ^3^. It shows a remarkable capacity to acquire and disseminate antimicrobial resistance determinants, rendering conventional therapies increasingly ineffective^1^, ^4^, ^5^. In recognition of this threat, the World Health Organization designated *A. baumannii* a critical priority pathogen in 2024, underscoring the need for novel therapeutics and innovative control strategies^6^. Beyond its well‐documented drug resistance, *A. baumannii* also uses a “persist‐and‐resist” strategy that enables survival under harsh conditions, including desiccation, disinfectants, oxidative stress, and host immune defenses^7^, ^8^, ^9^, ^10^. Recent studies have highlighted the importance of mobile genetic elements in facilitating bacterial adaptation to diverse environmental pressures by driving genome plasticity, including rearrangements and copy number variation^11^, ^12^, ^13^, ^14^. However, whether these genomic dynamics contribute to the “persist‐and‐resist” lifestyle of *A. baumannii* remains poorly understood.

Insertion sequences (ISs) are among the best‐characterized mobile elements capable of driving such genomic changes^11^, ^12^. ISs frequently catalyze such events by bracketing loci into translocatable units and promoting tandem duplication or copy number expansion^14^, ^15^. Most commonly, ISs‐mediated amplification has been reported for antimicrobial resistance genes (ARGs); for example, IS*26* or IS*CR1* can drive copy number increases of *bla*_TEM‐1_ and *bla*_NDM‐1_ in *Enterobacterales* under antibiotic selection, producing stepwise increases in resistance^14^, ^16^, ^17^. Our prior work showed that IS*26* can amplify multiple resistance regions, including *bla*_KPC‐2_, under selection, markedly enhancing multidrug resistance in clinical *Escherichia coli* isolates^18^. *A. baumannii* harbors numerous ISs, reflecting its strong potential for genomic rearrangement and adaptation^19^, ^20^. IS‐mediated amplification of aminoglycoside resistance genes has been frequently observed in this species^21^, ^22^. Among these elements, IS*Aba1* is the most characteristic IS in *A. baumannii* and can mediate the amplification and upregulation of the intrinsic *oxaAb* carbapenemase gene under carbapenem selection pressure^23^. However, the extent to which IS‐mediated amplifications influence genes beyond classical antibiotic resistance determinants, particularly those involved in host adaptation and stress response, remains poorly characterized.

In this study, we analyzed 7490 *A. baumannii* genomes and identified the IS*Aba1*‐flanked *crp‐osmC* module as a recurrently amplified locus. *crp* encodes the cAMP receptor protein (CRP), a global transcriptional regulator involved in multiple stress responses^24^, ^25^, ^26^, ^27^, whereas *osmC* encodes an osmotically inducible peroxidase‐like protein that protects against oxidative stress^28^. We found that amplification of the *crp*‐*osmC* module enhanced *A. baumannii* adaptation *in vitro* and *in vivo,* but compromised biofilm formation and antibiotic resistance.

## RESULTS

### Prevalence of *crp‐osmC* gene cluster amplification in *A. baumannii* genomes

To investigate the contribution of IS‐mediated gene amplification to the adaptability of *A. baumannii*, we analyzed 582 complete genomes available in the National Center for Biotechnology Information (NCBI) database as of May 2023, representing a diverse collection of global isolates. Using a custom Python script (available at GitHub: https://github.com/hailingfang/leaf), 1000 bp segments were extracted as seeds to identify homologous regions, and repeat boundaries were refined by iteratively extending the regions in 200 bp increments. Genes flanked by IS elements and present in multiple copies within individual genomes were identified, and candidate repeats were subsequently subjected to functional annotation to characterize IS‐mediated repeat elements (Figure S1A). Among the IS elements identified, IS*Aba1* was most frequently associated with gene amplification events (Table S1). The majority of amplified loci corresponded to ARGs, including *bla*_OXA‐23_, *aph(3')‐VI*, *sul2*, and others (Tables 1 and S1). Among these, *bla*_OXA‐23_ was the most frequently amplified, detected in 82 genomes (14.1%) (Table 1). The second most common amplification event involved a gene unit comprising *crp* and *osmC*, flanked by two IS*Aba1* elements, and was observed in 19 genomes (3.3%), with copy numbers ranging from 2 to 8 (Table 1 and Figure S1B).

**Table 1:** IS‐mediated gene amplification events observed in two or more of 582 complete *Acinetobacter baumannii* genomes.

Locus	**Number of genomes with amplification**	**Max copy number per genome**	**Major IS**
*bla*_OXA‐23_	82	5	IS*Aba1*
*crp‐osmC*	19	8	IS*Aba1*
*bla*_OXA_‐others	14	5	IS*Aba1*
*aph(3')‐VI*	11	14	IS*Aba125*
*bicA*	8	3	IS*Aba11*
*sul2*	6	2	IS*Aba1*
*eptA*	3	2	IS*Aba13*
*aph(3')‐Ia*	3	7	IS*26*
*aacC1*	2	3	IS*Aba15*
*xerC*	2	2	IS*26*

IS, insertion sequence.

The *crp‐osmC* gene cluster, a highly conserved element within the *A. baumannii* genome, is situated between the *akr* and *nat* genes, coding for NADPH‐dependent aldo‐keto reductase and N‐acetyltransferase, respectively (Figure S1B). IS*Aba1* elements flanking both ends of this gene unit form a composite transposon, potentially conferring copy‐and‐paste transposition. We designated this composite transposon as Tn*7691* in The Transposon Registry (https://transposon.lstmed.ac.uk/tn-registry) (Figure S1B). Analysis of insertion sites revealed that Tn*7691* consistently generated 9‐bp direct repeats enriched in TA sequences (Figure S1C). These events were not confined to a single locus but occurred at diverse genomic positions, with 13 distinct insertion sites identified across 19 complete genomes carrying amplifications (Table S2).

### Increased *crp‐osmC* copy number enhances bacterial growth

The CRP consists of two structural domains: a cyclic nucleotide‐binding domain and a helix–turn–helix (HTH) DNA‐binding domain. In our isolates, the IS*Aba1* was integrated at position PHE217 of the *crp* gene, corresponding to the terminal region of the HTH domain (Figure S2). This insertion adds three amino acids and introduces a premature stop codon. Structural predictions using AlphaFold2 and Phyre2 indicate that the modified CRP adopts a conformation similar to the native protein (CRP^wt^), suggesting that its functional integrity is largely retained (Figure S2). In addition, IS*Aba1* insertion downstream of the *osmC* gene did not affect its expression (Figure S2).

To investigate the biological significance of *crp‐osmC* gene cluster amplification, we cloned the “IS*Aba1‐crp‐osmC*” sequence into the pKFAb plasmid to mimic gene amplification and introduced the resulting construct into *A. baumannii* ATCC19606 by electroporation, generating the derivative strain 19606 (pKFAb:*crp*‐*osmC*) (Figure 1A). Subsequent quantitative PCR (qPCR) analysis confirmed a 6.23 ± 1.97‐fold increase in *crp* gene copy number in this strain (Figure 1B). In parallel, IS*Aba1*‐*crp* and *osmC* were individually cloned into the pKFAb plasmid and introduced into ATCC19606 by electroporation, generating the 19606 (pKFAb:*crp*) and 19606 (pKFAb:*osmC*) strains, which carried *crp* or *osmC* alone, respectively. The effects of this amplification on bacterial physiology were assessed by monitoring growth kinetics in Brain–Heart Infusion (BHI) medium. The strain 19606 (pKFAb:*crp‐osmC*) showed an enhanced growth phenotype compared with the control strain 19606 (pKFAb), reaching higher optical densities at later time points, with the difference becoming apparent mainly after approximately 12 h (Figure 1C). By contrast, amplification of either gene alone had no detectable effect on bacterial growth (Figure 1C). These findings demonstrate that amplification of the *crp‐osmC* gene cluster, rather than either gene individually, may contribute to improved stationary‐phase adaptation.

**Figure 1:**
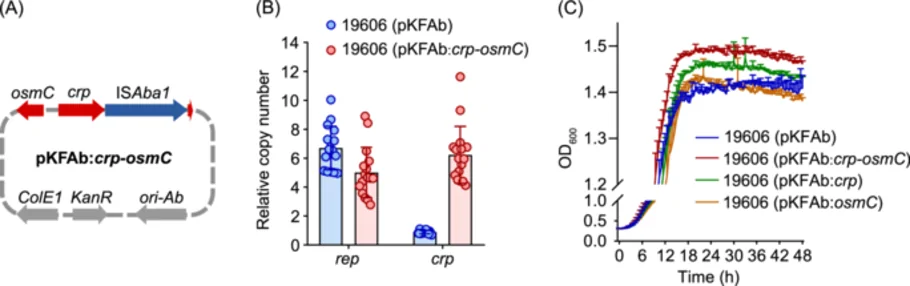
Simulation amplification of the *crp‐osmC* gene cluster improves stationary phase growth of *Acinetobacter baumannii*. (A) Structural design of plasmid pKFAb:*crp‐osmC*. (B) Quantification of gene copy numbers in *A. baumannii* strain ATCC19606 carrying pKFAb:*crp‐osmC* or the empty vector pKFAb (*n* = 16). (C) Growth curves of *A. baumannii* ATCC19606 derivative strains carrying pKFAb, pKFAb:*crp*, pKFAb:*osmC,* or pKFAb:*crp*‐*osmC* in Brain–Heart Infusion (BHI) medium (*n* = 5). *rep* refers to the gene encoding the plasmid replication protein.

### Elevated *crp‐osmC* copy number enhances *in vivo* colonization and promotes serum resistance

To further evaluate the competitive fitness of the amplified strain *in vitro* and its colonization potential *in vivo*, we incorporated a red fluorescent protein marker (mCherry) into the empty vector as control strain 19606 (pKFAb) to enable direct differentiation during co‐culture and infection assays. For *in vitro* competition assays, equal ratios of 19606 (pKFAb:*crp‐osmC*) and 19606 (pKFAb:mCherry) strains were co‐cultured in liquid medium, and bacterial counts were determined at 3 and 6 h. Compared with the 19606 (pKFAb:mCherry) strain, 19606 (pKFAb:*crp‐osmC*) displayed a competitive advantage, with competitive index values of 3.06 ± 0.88 and 3.45 ± 0.67 at 3 and 6 h, respectively (Figure 2A). We next evaluated whether the observed advantages extend to a living host. Using murine lung infection and intestinal colonization models, mice were intranasally or orally inoculated with a 1:1 mixture of the two strains (Figure 2B). At 24 h post inoculation (hpi), body weight was recorded, an relative bacterial loads in lung tissue or faecal samples were quantified (Figures 2B–D and S3). Strain 19606 (pKFAb:*crp‐osmC*) consistently outcompeted 19606 (pKFAb:mCherry) in both pulmonary (competitive index of 3.56 ± 0.97) and intestinal (competitive index of 2.12 ± 0.79) niches, demonstrating a clear competitive advantage at 24 hpi (Figure 2C,D).

**Figure 2:**
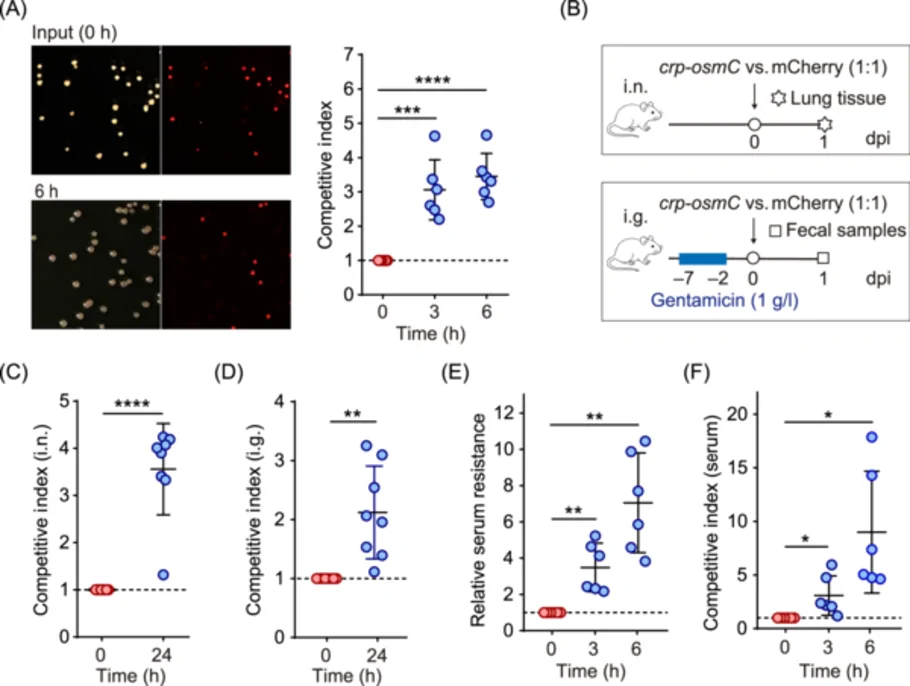
*crp‐osmC* amplification confers a competitive advantage *in vitro*, enhances colonization in murine models, and increases serum resistance. (A) *In vitro* competition between ATCC19606 (pKFAb:mCherry) and ATCC19606 (pKFAb:*crp‐osmC*) mixed at a 1:1 ratio in BHI medium. Data are presented as mean ± SD (*n* = 6). One‐way ANOVA followed by Tukey's multiple comparisons test was performed (***p < 0.001; and ****p < 0.0001). (B) Schematic of mouse infection models: intranasal (i.n.) for pulmonary infection and intragastric (i.g.) for intestinal colonization (*n* = 8 per group). Both models received 2 × 10^8^ CFU ATCC19606 (pKFAb:mCherry) or ATCC19606 (pKFAb:*crp‐osmC*). For the intestinal infection model, mice were pretreated with gentamicin (1 g/l in drinking water for 5 days) before inoculation. Bacterial ratios were determined from lung or fecal samples 24 h post infection. (C, D) Competitive index of strains in mouse lungs (C) and feces (D). At 24 h post infection, samples were harvested, homogenized, and plated on Luria–Bertani (LB) agar supplemented with kanamycin. CFU counts of red fluorescent (pKFAb:mCherry) and non‐fluorescent (pKFAb:*crp‐osmC*) strains were enumerated, and the competitive index between the two populations was calculated. Data are presented as mean ± SD (*n* = 8). One‐sample *t*‐test against the hypothetical value of 1.0 was performed. (E) Serum resistance of ATCC19606 (pKFAb:*crp‐osmC*) relative to ATCC19606 (pKFAb:mCherry). (F) Competition between the two strains mixed at a 1:1 ratio in mouse serum. Data in (E) and (F) are presented as mean ± SD (*n* = 6). Two‐tailed, paired *t*‐test was performed. (**p < 0.01; ***p < 0.001; and ****p < 0.0001). ANOVA, analysis of variance.

We next measured resistance to serum‐mediated killing, an important determinant of host‐associated fitness. The amplified strain 19606 (pKFAb:*crp‐osmC*) displayed increased survival in mouse serum, with a 3.49 ± 1.34‐ and 7.05 ± 2.75‐fold increase over strain 19606 (pKFAb:mCherry) at 3 and 6 h, respectively (Figure 2E). Moreover, when the two strains were co‐incubated in mouse serum at a 1:1 ratio, competition assays yielded consistent results, with competitive index values of 3.09 ± 1.84 and 9.00 ± 5.69 for strain 19606 (pKFAb:*crp‐osmC*) at the same time points (Figure 2F).

To further assess whether the pulmonary competitive advantage was dependent on cyclophosphamide‐sensitive cellular immune pressure, we performed an additional pulmonary competition assay in cyclophosphamide‐treated mice. Mice were intraperitoneally administered cyclophosphamide at 150 and 100 mg/kg on Day 3 and 1 before infection, respectively, followed by intranasal inoculation with a 1:1 mixture of 19606 (pKFAb:*crp‐osmC*) and 19606 (pKFAb:mCherry) strains on Day 0 (Figure S4A). Body weight was recorded at 0 and 24 hpi, and bacterial loads in lung tissues were quantified at 24 hpi (Figure S4B and S4C). Strain 19606 (pKFAb:*crp‐osmC*) retained a competitive advantage over 19606 (pKFAb:mCherry), with a competitive index of 5.54 ± 1.98 at 24 hpi (Figure S4C). These results indicate that the pulmonary fitness advantage conferred by *crp‐osmC* amplification is independent of cyclophosphamide‐sensitive cellular immune pressure.

### Transcriptomic analysis identifies metabolic and siderophore‐related changes associated with *crp‐osmC* amplification

To investigate the impact of *crp*‐*osmC* amplification on bacterial physiology, we analyzed the transcriptomic profiles of 19606 (pKFAb) and 19606 (pKFAb:*crp*‐*osmC*) strains. Using the thresholds of false discovery rate (FDR) ≤ 0.01 and fold‐change (FC) ≥ 2, a total of 65 genes were upregulated and 45 genes were downregulated in 19606 (pKFAb:*crp‐osmC*) relative to 19606 (pKFAb) (Figure 3A). Kyoto Encyclopedia of Genes and Genomes (KEGG) enrichment analysis identified the three most significantly enriched pathways as phenylalanine metabolism, biosynthesis of siderophore group nonribosomal peptides, and sulfur metabolism (Figure 3B). Heatmap analysis showed that genes within these pathways were predominantly upregulated (Figure 3C). Eight genes within the *paa* gene cluster involved in phenylalanine metabolism were upregulated (Figure 3C). The *paa* pathway mediates phenylacetic acid degradation, generating intermediates that can feed into central carbon metabolism, including acetyl‐CoA and succinyl‐CoA (Figure 3D). Upregulation of this pathway may broaden carbon source utilization and enhance metabolic flexibility under host‐associated nutrient‐limited conditions, potentially contributing to the competitive fitness advantage of 19606 (pKFAb:*crp‐osmC*)^29^. In addition to the *paa* cluster, five siderophore biosynthesis‐related genes and four of five sulfur metabolism‐related genes were significantly upregulated (Figure 3C). Because iron acquisition is critical for *A. baumannii* growth under host‐imposed nutrient limitation, upregulation of siderophore‐associated genes may enhance bacterial adaptation during infection^30^. Sulfur metabolism may also contribute to nutrient utilization and stress adaptation^31^, ^32^. These transcriptomic changes indicate that *crp‐osmC* amplification is associated with coordinated upregulation of pathways involved in metabolic flexibility and nutrient acquisition.

**Figure 3:**
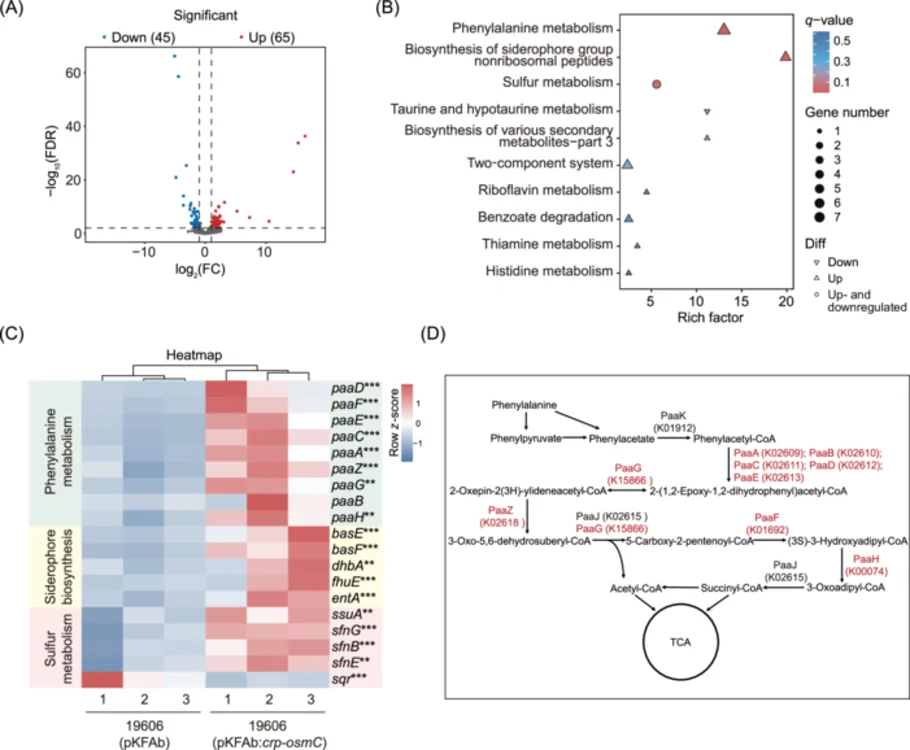
Transcriptomic analysis of *crp‐osmC* amplification. (A) Volcano plot of differentially expressed genes in 19606 (pKFAb:*crp‐osmC*) versus 19606 (pKFAb). (B) Kyoto Encyclopedia of Genes and Genomes (KEGG) pathway enrichment analysis of differentially expressed genes in 19606 (pKFAb:*crp‐osmC*) relative to 19606 (pKFAb). (C) Heatmap showing differentially expressed genes associated with phenylalanine metabolism, biosynthesis of siderophore group nonribosomal peptides, and sulfur metabolism. Row‐scaled FPKM values are shown as *z*‐scores using a blue–white–red color scale ranging from –1 to 1. 1, 2, and 3 represent three independent biological replicates. Stars next to gene names indicate FDR‐adjusted significance: ***p < 0.001, **p < 0.01, and *p < 0.05. (D) KEGG‐based phenylalanine/phenylacetate degradation network. Upregulated genes within the *paa* cluster identified in 19606 (pKFAb:*crp‐osmC*) were mapped to KEGG phenylalanine metabolism and phenylacetate degradation pathways. The *paa*‐associated degradation network was drawn based on KEGG metabolic maps, illustrating the conversion of phenylalanine‐derived phenylacetate/phenylacetyl‐CoA into acetyl‐CoA and succinyl‐CoA entering the tricarboxylic acid (TCA) cycle. Upregulated *paa* genes are highlighted in red, and identifiers in parentheses represent the corresponding KEGG Orthology numbers.

To determine whether CRP directly regulates these transcriptionally altered gene clusters, we performed electrophoretic mobility shift assays (EMSAs) using purified CRP protein (encoded by the IS*Aba1*‐interrupted *crp* allele), with CRP^wt^ included as a control (Figure S5A). Promoter regions of the *paa*, *bas*, and *sfn* clusters were selected for DNA‐binding assays. Both CRP and CRP^wt^ bound to the *bas* promoter (Figure S5B), whereas no obvious binding was detected for the *paa* or *sfn* promoters (Figure S5C,D). These results suggest that CRP retains DNA‐binding activity and may directly regulate siderophore‐associated *bas* genes, whereas upregulation of *paa* and *sfn* genes appears to be mediated indirectly.

### *crp‐osmC* amplification is associated with altered biofilm formation

In addition to the enrichment of pathways related to phenylalanine metabolism and siderophore biosynthesis, Gene Ontology (GO) biological process analysis also identified enrichment of genes associated with pilus assembly and biofilm formation (Figure 4A). We therefore extracted the biofilm‐related differentially expressed genes for further analysis. Most of these genes were downregulated in 19606 (pKFAb:*crp‐osmC*) compared with 19606 (pKFAb), including six genes from the *csu* pilus cluster, four genes from the *mrk*‐like fimbrial cluster, and three genes from the *pga* cluster (Figure 4B). The *csu* cluster is involved in chaperone–usher pilus assembly and initial attachment to abiotic surfaces^33^, whereas the *mrk*‐like cluster encodes fimbrial components that may contribute to surface adhesion and biofilm development^34^. The *pga* cluster is responsible for the biosynthesis and export of poly‐β‐1,6‐N‐acetyl‐d‐glucosamine, an important exopolysaccharide component of the biofilm matrix^35^. Together, the coordinated downregulation of these genes indicates that *crp‐osmC* amplification impairs surface‐associated biofilm formation. To determine whether CRP directly interacts with the promoters of these biofilm‐associated gene clusters, we performed EMSAs using promoter regions of the *csu*, *mrk*‐like, and *pga* clusters. CRP bound to the promoter regions of the *csu* and *pga* clusters (Figure 4C), whereas no obvious binding was detected for the *mrk*‐like promoter region (Figure S6). These findings demonstrate that CRP may directly participate in the transcriptional regulation of biofilm‐associated genes *csu* and *pga*.

**Figure 4:**
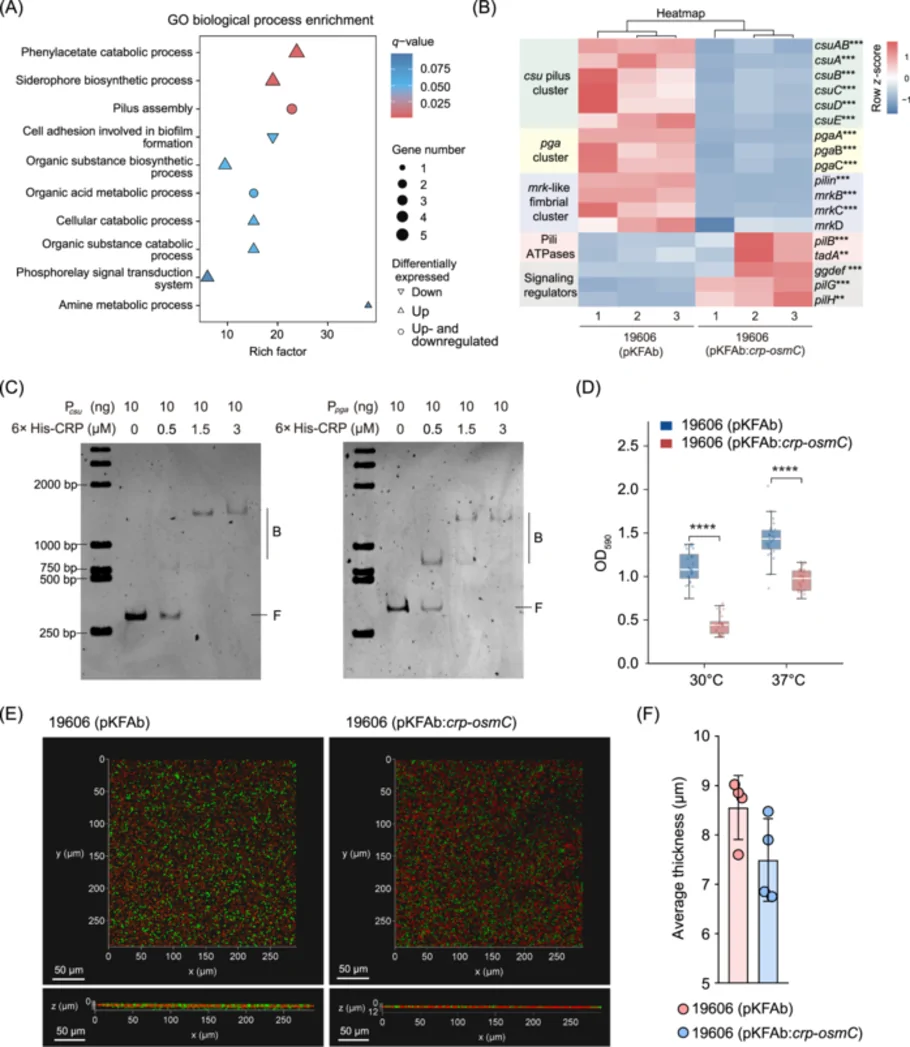
*crp‐osmC* amplification is associated with reduced biofilm formation. (A) Gene Ontology (GO) biological process enrichment analysis of differentially expressed genes in 19606 (pKFAb:*crp‐osmC*) compared with 19606 (pKFAb). (B) Heatmap of differentially expressed genes associated with pilus assembly and biofilm formation. (C) Electrophoretic mobility shift assay (EMSA) analysis of the binding activity of CRP toward the putative promoter regions of the *csu* and *pga* clusters. B, bound probe; F, free probe. (D) Quantification of biofilm biomass by crystal violet staining after 24 h of static incubation at 30°C or 37°C. Data are shown as mean ± SEM; *n* = 24 technical replicate wells per group. Two‐tailed unpaired Student's *t*‐test; ****p < 0.0001. (E) Representative confocal microscopy images of pellicle‐like biofilms formed at the air–liquid interface after staining with SYTO 9 and propidium iodide. Scale bars, 50 μm. (F) Quantification of average biofilm thickness from confocal Z‐stack images using ImageJ. Data are shown as mean ± SD; *n* = 4 fields.

To test whether these transcriptional changes were associated with altered biofilm formation, we compared the biofilm‐forming capacities of 19606 (pKFAb) and 19606 (pKFAb:*crp‐osmC*) using crystal violet staining. Biofilm production was measured after 24, 48, and 72 h of incubation at both 30°C and 37°C. At 24 h, 19606 (pKFAb:*crp‐osmC*) showed reduced biofilm formation compared with 19606 (pKFAb) at both temperatures (Figure 4D). To corroborate the crystal violet staining results and visualize the architecture of pellicle‐like biofilms, 24 h biofilms were stained with SYTO 9 and propidium iodide and analyzed by confocal microscopy. Three‐dimensional reconstruction of *Z*‐stack images showed that biofilms formed by 19606 (pKFAb:*crp‐osmC*) appeared less compact than those formed by 19606 (pKFAb) (Figure 4E). Quantitative analysis showed a modest decrease in average biofilm thickness, from 8.56 ± 0.65 μm in 19606 (pKFAb) to 7.50 ± 0.48 μm in 19606 (pKFAb:*crp‐osmC*) (Figure 4F). We next examined whether this reduction persisted at later time points. At 48 and 72 h, 19606 (pKFAb:*crp‐osmC*) continued to produce less biofilm than 19606 (pKFAb) at 30°C (Figure S7). However, at 37°C, no significant difference in biofilm formation was observed between 19606 (pKFAb) and 19606 (pKFAb:*crp‐osmC*) at these later time points (Figure S7). These results demonstrate that *crp‐osmC* amplification is associated with reduced biofilm formation in a time‐ and temperature‐dependent manner.

### Gain of *crp‐osmC* copy number elevates antibiotic sensitivity in *A. baumannii*

To investigate the effect of *crp*‐*osmC* gene cluster amplification on antibiotic susceptibility in *A. baumannii*, we performed serial dilution assays with cultures exposed to a spectrum of antibiotic classes. Contrary to expectations, amplification of the *crp‐osmC* gene cluster increased sensitivity to a broad range of antibiotics (Figure 5). This heightened sensitivity spanned four major antibiotic classes: *β*‐lactams (including meropenem, biapenem, ertapenem, ampicillin, ceftazidime, and aztreonam), polypeptides (polymyxin B), fluoroquinolones (ciprofloxacin), and phenicols (chloramphenicol). The amplification did not uniformly affect all antibiotics tested; tetracycline and fosfomycin showed no substantial change in efficacy against the amplified strains, indicating a selective impact of gene amplification on antibiotic sensitivity (Figure 5).

**Figure 5:**
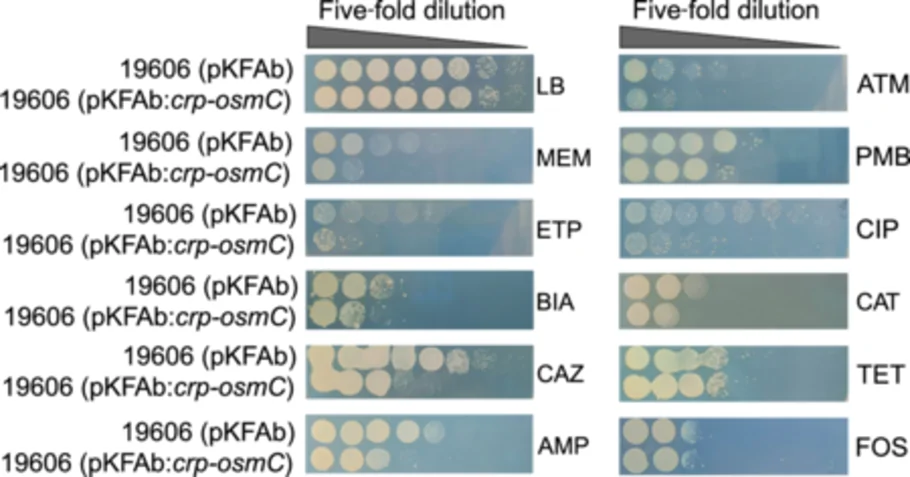
*crp‐osmC* amplification selectively increases sensitivity to multiple antibiotic classes. Spot dilution assays on LB agar without antibiotics (LB) or supplemented with antibiotics at the indicated concentrations: Ampicillin (AMP, 800 mg/l), Aztreonam (ATM, 16 mg/l), Biapenem (BIA, 0.8 mg/l), Ceftazidime (CAZ, 5 mg/l), Chloramphenicol (CAT, 128 mg/l), Ciprofloxacin (CIP, 1 mg/l), Ertapenem (ETP, 8 mg/l), Fosfomycin (FOS, 100 mg/l), Meropenem (MEM, 2 mg/l), Polymyxin B (PMB, 2 mg/l), and Tetracycline (TET, 6 mg/l).

To further investigate the role of the *crp‐osmC* gene cluster in antibiotic susceptibility, we constructed a double‐knockout mutant, Δ*crp*Δ*osmC*. Compared with the wild‐type strain, this mutant displayed decreased antibiotic sensitivity (Figure S8), indicating that the *crp*‐*osmC* gene cluster contributes to a regulatory network that limits the antibiotic tolerance. These results demonstrate a dosage‐dependent effect, with amplification increasing antibiotic sensitivity and deletion enhancing tolerance.

Previous studies have implicated the *crp* and *osmC* genes in the regulation of various stress responses, including oxidative stress, osmotic pressure, and ultraviolet (UV) radiation. However, resistance assays revealed that amplification of the *crp‐osmC* gene cluster did not alter resistance to oxidative agents (such as H_2_O_2_ and cumene hydroperoxide), UV radiation, osmotic stress (induced by sodium chloride), or disinfectants (including chlorhexidine acetate and benzalkonium chloride) (Figure S9). These results suggest that the observed modulation of antibiotic susceptibility is independent of the canonical stress response pathways.

### IS*Aba1*‐mediated *crp‐osmC* amplification is predominantly associated with ST2 clinical *A. baumannii*

To further investigate the distribution and lineage association of IS*Aba1*‐mediated *crp*‐*osmC* amplification, we expanded our dataset by incorporating an additional 6908 draft genomes, which were analyzed together with the 582 complete genomes (Figure S1A). Our analysis showed that 35.9% (2689 of 7490) of the genomes harbored IS*Aba1* insertions within the *crp* gene, including 250 complete genomes and 2439 draft genomes (Figure S10A). The earliest recorded IS*Aba1* insertion in *crp* dates to 1996 (Figure S10B). Among these IS*Aba1*‐in‐*crp*‐positive genomes, Tn*7691* accounted for 11.2% (300/2689) (Figures S10A,B). These observations are consistent with a stepwise process involving IS*Aba1* insertion, Tn*7691* formation, and subsequent amplification, with the emergence of Tn*7691* providing the genetic basis for *crp‐osmC* amplification. The 300 Tn*7691*‐positive genomes (271 draft genomes and 29 complete genomes) were subjected to phylogenetic analysis incorporating associated metadata, including year of isolation, source, sequence types, and region (Figure 6). These strains from six regions were evenly distributed between 2005 and 2021, and predominantly derived from clinical isolates (216 of 227 strains with available source information) (Figures 6 and S10C). Of the 300 strains, 292 belonged to Sequence Type 2 (ST2), while the remaining eight were ST2‐ Single Locus Variant (SLV) (Figure 6). ST2 is a globally prevalent high‐risk clone of *A. baumannii*, closely linked to multidrug resistance, especially OXA‐23‐mediated carbapenem resistance, and heightened virulence^2^, ^36^. Among the 271 draft genomes, only 179 had raw sequencing data available in the Sequence Read Archive (SRA), enabling precise identification of 55 amplification events through read mapping against a reference genome (Figures 6 and S10A). Together with 19 amplification strains from complete genomes, a total of 74 strains showed *crp‐osmC* amplification, with copy numbers ranging up to eight, most commonly ranging from 1.5 to 3 copies (Figures 6 and S10A). Strains from the United States accounted for 70% of all documented amplification events (Figures 6 and S10C). All Tn*7691* amplification events with available source information were identified in clinical isolates, highlighting the strong association of these genetic changes with host‐associated fitness in *A. baumannii* (Figure 6).

**Figure 6:**
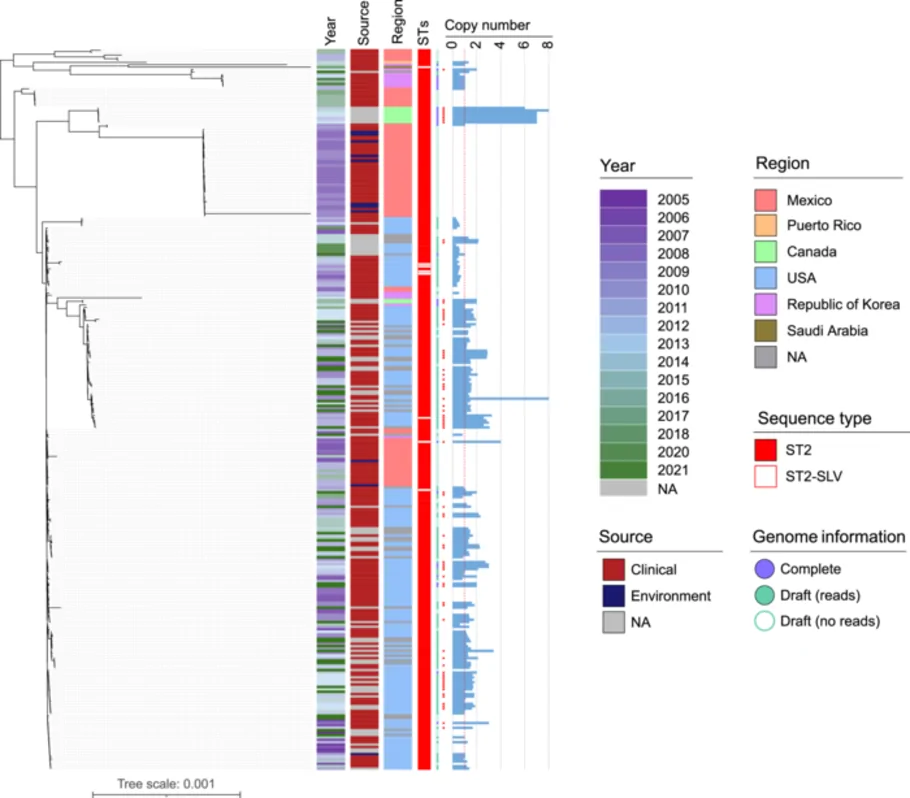
*crp‐osmC* amplification is predominantly associated with clinical isolates of the globally disseminated ST2 lineage. Phylogenetic tree of 300 *A. baumannii* genomes carrying Tn*7691* (271 draft and 29 complete), annotated with isolation year, source, region, sequence types (STs), and copy numbers is shown. Red pentagrams indicate 74 isolates showing amplification, identified in complete genomes (copy number ≥2) and draft genomes with sequencing read support (depth ratio ≥1.5). NA, not available.

To determine whether *crp‐osmC* amplification also confers a competitive advantage in clinical ST2 backgrounds, we constructed pKF‐ble:*crp‐osmC*‐bearing derivatives of two clinical ST2 isolates: 21B076 and 21C108. Increased *crp‐osmC* copy number did not noticeably affect monoculture growth in BHI at 37°C; however, both pKF‐ble:*crp‐osmC*‐bearing derivatives outcompeted their corresponding empty vector controls in *in vitro* competition assays (Figure S10D), indicating that this competitive advantage is not restricted to the ATCC19606 laboratory background. Because the two ST2 clinical isolates showed high‐level resistance to multiple antibiotics, their resistant backgrounds limited the ability of spot dilution assays to resolve additional changes in antibiotic susceptibility. We therefore introduced pKFAb:*crp‐osmC* into a relatively antibiotic‐susceptible clinical isolate, XH858. Compared with XH858 (pKFAb), XH858 (pKFAb:*crp‐osmC*) showed reduced growth on Luria–Bertani (LB) agar supplemented with meropenem or ceftazidime (Figure S10E), indicating that *crp‐osmC* amplification confers a competitive benefit in clinical *A. baumannii* backgrounds while increasing susceptibility to these antibiotics.

## DISCUSSION

*A. baumannii* is known for its exceptional adaptability and has evolved to flourish in a variety of environments, which augments its transmission and colonization potential. The genetic underpinnings of *A. baumannii*'s adaptability are not fully understood. Our study provides evidence that amplification of the *crp*‐*osmC* locus, facilitated by IS*Aba1*, represents an evolutionary adaptation in the globally disseminated ST2 lineage. This genetic modification enhances host‐associated fitness, as shown by improved competitive growth, serum resistance, and colonization efficiency in mouse models.

Gene amplification in bacteria facilitates adaptation to fluctuating environments, particularly under antibiotic pressure, by rapidly generating variable copy numbers and expression polymorphisms^11^, ^12^. IS elements play a central role in promoting this rapid amplification process^18^. Analysis of genomic data revealed the widespread occurrence of IS‐mediated amplification of ARGs in *A. baumannii*, including those conferring resistance to oxacillin, sulfonamides, aminoglycosides, tetracyclines, macrolides, and other antibiotic classes (Table S1). Among these, IS*Aba1* was identified as the predominant mediator. These findings expand current understanding of the genomic plasticity of *A. baumannii*, highlighting gene amplification as an important yet previously underappreciated mechanism contributing to its adaptive evolution under antibiotic pressure.

Escaping the immune system's attacks is a crucial factor contributing to *A. baumannii* adaptability and is widely regarded as a major determinant of its pathogenicity^8^, ^37^. Our data show that *crp‐osmC* amplification increased *in vivo* competitive fitness and serum resistance, indicating that this locus contributes to host‐associated adaptation. Transcriptomic analysis suggest that the enhanced host‐associated fitness of the amplified strain may be linked to changes in metabolic and nutrient acquisition pathways, particularly phenylalanine metabolism, siderophore biosynthesis, and sulfur metabolism. Metabolic adaptation is increasingly recognized as an important determinant of *A. baumannii* infection biology and antimicrobial responses^38^. Upregulation of the *paa* pathway may broaden carbon source utilization by feeding phenylacetic acid catabolism into central carbon metabolism, thereby improving metabolic flexibility under nutrient‐limited host‐associated conditions. In parallel, increased expression of siderophore biosynthesis genes may enhance iron acquisition during infection, where free iron is restricted by the host^30^. CRP‐family regulators are typically involved in coordinating carbon metabolism with broader physiological responses, and cAMP‐associated signaling has been implicated in *A. baumannii* virulence regulation and adaptive antibiotic resistance^24^, ^39^. In our study, CRP retained DNA‐binding activity and bound to the promoter region of the siderophore‐associated *bas* cluster, supporting a direct role in regulating iron acquisition‐related genes. In contrast, the absence of detectable CRP binding to the *paa* or *sfn* promoters suggests that their upregulation is more likely mediated indirectly through downstream regulatory or metabolic changes associated with *crp‐osmC* amplification. The adjacent *osmC* gene may further contribute to stress adaptation, as OsmC‐family proteins are associated with protection against peroxide and osmotic stress^28^. Together, concurrent amplification of *crp* and *osmC* may provide a combined advantage by coupling transcriptional regulation with enhanced stress resilience. However, we noted that the transcriptomic changes occurred in a laboratory‐adapted strain under *in vitro* conditions; whether similar regulatory patterns occur during infection requires further investigation.

Our findings show that amplification of the *crp*‐*osmC* locus enhanced host‐associated fitness while modestly increasing antibiotic susceptibility, revealing a potential adaptive trade‐off. This trade‐off may be linked to altered carbon metabolism, which could reduce the prevalence of dormant or antibiotic‐tolerant subpopulations, consistent with the close association between bacterial persistence and metabolic state^40^. Additionally, *crp*‐*osmC* amplification downregulated the *csu*, *mrk*‐like, and *pga* genes and reduced biofilm biomass and thickness, potentially compromising matrix‐mediated protection, surface attachment, and collective tolerance^41^.

*A. baumannii* ST2 represents the most prevalent and clinically significant lineage among carbapenem‐resistant *A. baumannii* (CRAB)^42^. Large‐scale genomic and epidemiological studies have shown that ST2 accounts for over 60% of all sequenced CRAB isolates worldwide and has been reported in more than 100 countries^2^, ^36^, ^42^. This lineage predominates in clinical settings and is strongly associated with the dissemination of *bla*_OXA‐23_ and other carbapenemase genes, contributing to its exceptional multidrug resistance and global persistence^36^. In our study, all strains harboring Tn*7691* or showing *crp‐osmC* amplification belonged to ST2 or its single‐locus variant (ST2‐SLV), underscoring the tight genetic linkage between this amplification event and the dominant epidemic clone.

Collectively, our findings identify IS*Aba1*‐mediated *crp*‐o*smC* amplification as a genetic mechanism that may have contributed to the adaptive success of the globally disseminated ST2 lineage. Several limitations should nevertheless be considered. First, the transcriptomic analysis was performed in the laboratory‐adapted strain ATCC19606 under *in vitro* conditions; whether the same regulatory patterns occur in clinical isolates during infection remains to be determined. Second, although we demonstrated a correlation between *crp‐osmC* amplification and altered antibiotic susceptibility, the precise molecular mechanisms linking amplification to specific antibiotic sensitivity phenotypes remain to be fully elucidated.

## MATERIALS AND METHODS

### Bioinformatics analysis of IS‐mediated repeat region copy numbers in *A. baumannii* strains

We developed custom Python scripts to mine the complete genomes of 582 *A. baumannii* strains for repeat regions (https://github.com/hailingfang/leaf). Regions were defined as repeats if they showed ≥ 90% identity and ≥90% coverage within a single strain. Only repeat regions exceeding 1000 bp in length were considered. The identified repeat regions were then aligned with the ISfinder database to filter out those containing ISs^43^. A repeat region was excluded if the length of the IS occupied more than 70% of the entire repeat. Gene prediction within IS‐containing repeat regions was performed using Prokka^44^. ARGs were detected by homology‐based search against the Comprehensive Antibiotic Resistance Database (CARD)^45^.

### Analysis of IS*Aba1*‐mediated amplification of the *crp‐osmC* gene in *A. baumannii*

To gain a deeper insight into the amplification of the *crp‐osmC* gene mediated by IS*Aba1*, we examined the whole‐genome sequences of 7490 *A. baumannii* strains, which included 582 complete and 6908 draft genomes, all of which were retrieved from the NCBI database. Additionally, raw sequencing data for 179 draft genomes were obtained from the Sequence Read Archive (SRA). Each genome was subjected to a homology‐based search to detect the presence of IS*Aba1*. We specifically quantified the instances where IS*Aba1* was inserted within or adjacent to the *crp* gene. Subsequently, we sought to identify the transposon Tn*7691*. For complete genomes, the copy numbers of Tn*7691* were determined directly from the homology alignment results. For draft genomes, we estimated the copy numbers by mapping reads and calculating the ratio of the average read depth across the Tn*7691* region to the average read depth of the entire genome. We extracted metadata, including the isolation time, the geographical location, and the source of each strain, from their respective GenBank (gbk) files. The direct repeats associated with Tn*7691* were extracted from the genome sequences, and a consensus analysis of their sequences was performed using WebLogo^46^. Sequence types (STs) were identified via the pubMLST database^47^. We constructed a phylogenetic tree based on the core genes of the 300 *A. baumannii* genomes containing Tn*7691*, which was refined using iTOL and Inkscape (version 0.92) for enhanced visual representation.

### Bacterial strains, culture, and antibiotics

The bacterial strains and plasmids used in this study are listed in Table S3. All *A. baumannii* strains and mutants were grown aerobically at 37°C in BHI medium with shaking at 200 rpm. Colony formation was on LB agar at 37°C. The antibiotics kanamycin (50 mg/l) and bleomycin (200 mg/l) were added for selection as needed.

### Simulated gene amplification and gene deletion

To simulate the amplification of the *crp‐osmC* gene and IS*Aba1* insertion as observed in natural settings, we cloned the IS*Aba1‐crp‐osmC* sequence into the pKFAb plasmid, generating the construct pKFAb:*crp‐osmC*. This plasmid was introduced into *A. baumannii* ATCC19606 by electroporation, resulting in strains carrying amplified copies of *crp‐osmC*. In parallel, pKFAb:mCherry and the empty vector pKFAb were electroporated into ATCC19606 as controls. To construct a *crp‐osmC* deletion in *A. baumannii* ATCC19606, the pK18mobsacB–Δ*crp*Δ*osmC* plasmid was transformed into *A. baumannii* ATCC19606 by electroporation and integrated into the chromosome via homologous recombination to achieve single crossover^48^, ^49^. Transconjugants were selected on LB agar containing 50 mg/l kanamycin. For markerless in‐frame deletion, counter‐selection was performed on LB agar containing 15% sucrose. We screened for strains that could grow on sucrose but were sensitive to kanamycin by parallel plating on LB agar with 50 mg/l kanamycin and 15% sucrose. Deletion was confirmed by PCR amplification using the UP‐F/DO‐R primer pair (Table S4) and DNA sequencing.

To assess whether amplification of *crp‐osmC* confers a similar phenotype in clinical strains, the kanamycin resistance cassette in pKFAb was replaced with a bleomycin resistance cassette by homologous recombination, yielding pKF‐ble and pKF‐ble:*crp‐osmC*. The primers used to construct pKF‐ble:*crp‐osmC* are listed in Table S4. Plasmids pKF‐ble and pKF‐ble:*crp‐osmC* were introduced into two *A. baumannii* ST2 clinical isolates, 21B076 and 21C108, whereas pKFAb and pKFAb:*crp‐osmC* were introduced into the clinical isolate XH858.

All electroporation procedures were performed using a Gene Pulser Xcell system (Bio‐Rad) at 1.8 kV, 200 Ω, and 25 μF.

### Determination of gene copy numbers through qPCR

To quantify the copy number variations of *crp‐osmC* and the plasmid replicon (*rep*), we performed qPCR using the $2^{‐∆C_{t}}$ method. Relative copy numbers were calculated by normalizing against the single‐copy housekeeping gene *rpoB*. Primers specific for *rpoB*, *crp*, and *rep* were designed using the IDT online tool (https://sg.idtdna.com/), with the sequences listed in Table S4. qPCR assays were carried out with the ChamQ Universal SYBR qPCR Master Mix (Vazyme Biotech) on an Applied Biosystems ViiA7 Real‐Time PCR System. Each experiment included 16 biological replicates and three technical replicates, and the mean *C*_t_ values were determined from replicates with a *C*_t_ SD < 0.3.

### Growth curves

To evaluate the effect of *crp‐osmC* amplification on bacterial growth, we recorded growth curves using an automated growth curve analyzer (Bioscreen C). Single colonies of *A. baumannii* ATCC 19606 carrying pKFAb, pKFAb:*crp*, pKFAb:*osmC*, or pKFAb:*crp‐osmC* were inoculated into BHI broth and cultured overnight at 37°C with shaking at 180 rpm. Each culture was then diluted 1:100 in fresh BHI broth, and a 200 μl aliquot was transferred to a 100‐well honeycomb microplate (Bioscreen, 9502550). Kanamycin (50 mg/l) was added to maintain plasmid stability. OD_600_ values were recorded every 30 min with continuous shaking over 48 h. Growth curves of the clinical ST2 strains were measured using the same procedure. Five replicates were performed per group, and growth curves were analyzed using GraphPad Prism.

### *In vitro* and *in vivo* competition experiments

For *in vitro* competition assays, we compared the relative fitness of 19606 (pKFAb:*crp‐osmC*) with 19606 (pKFAb:mCherry). All experiments included at least six biological replicates. Overnight cultures were diluted to 5 × 10^6^ colony‐forming units (CFU)/ml in BHI broth, mixed at a 1:1 ratio, and incubated at 37°C for 6 h. Samples taken at 0, 3, and 6 h were serially diluted and plated on LB agar with kanamycin. Colony ratios were determined using a LUYOR‐3421 benchtop fluorescent protein excitation source and further confirmed by PCR with IS*Aba1*‐F/IS*Aba1*‐R primers (Table S4). For competition assays in ST2 clinical isolates, paired strains carrying pKF‐ble or pKF‐ble:*crp‐osmC* were mixed at an approximately 1:1 ratio and co‐cultured in BHI at 37°C. Samples were collected at 0 and 6 h, serially diluted, and plated on LB agar with bleomycin. For each plate, 20–24 single colonies were randomly selected for PCR identification using cCRP‐test‐F/ISAba1‐R primers, and the relative abundance of each strain was calculated from PCR‐confirmed colony identities (Table S4). Five biological replicates were performed for each competition assay.

To construct an *in vivo* pulmonary infection model, female BALB/c mice (6–8 weeks old, *n* = 8) were used. Single colonies of 19606 (pKFAb:*crp‐osmC*) and 19606 (pKFAb:mCherry) were cultured in BHI broth at 37°C with shaking to an OD_600_ of ~1.0, washed with PBS, and adjusted to 1 × 10^10^ CFU/ml. A 1:1 mixture (20 μl, ~2 × 10^8^ CFU) was intranasally inoculated into mice under isoflurane anesthesia. At 24 hpi, body weight was recorded. Mice were euthanized in accordance with approved animal protocols, and the lungs were collected, homogenized, and plated on LB agar with kanamycin for CFU counts. For pulmonary competition assays in cyclophosphamide‐treated mice, immunosuppression was induced by intraperitoneal administration of cyclophosphamide monohydrate (Sigma‐Aldrich) at 150 mg/kg and 100 mg/kg on Day 3 and 1 before infection, respectively^50^. Mice were then intranasally inoculated with the 1:1 bacterial mixture as described above (*n* = 15). At 24 hpi, body weight was recorded, and lung tissues were collected for bacterial load determination and competitive index analysis.

To construct an *in vivo* intestinal colonization model, mice (*n* = 8) received gentamicin (1 g/l) in drinking water from Day –7 to Day –2, followed by antibiotic‐free water until Day 0^50^. On Day 0, a 1:1 mixture (100 μl, ~2 × 10^8^ CFU) of the two strains was administered intragastrically. At 24 h, body weight was recorded and fecal samples were collected, homogenized, and plated on LB agar with kanamycin. Strain ratios were quantified as described for *in vitro* assays.

### RNA‐seq and bioinformatic analysis

*A. baumannii* ATCC 19606 carrying pKFAb or pKFAb:*crp‐osmC* was cultured overnight and re‐inoculated into liquid BHI medium at an OD_600_ of 0.5 with 50 mg/l kanamycin. Cultures were grown for 2–4 h before RNA extraction. Total RNA was extracted using the High‐Purity RNA Extraction Kit (CWBIO) according to the manufacturer's protocol. RNA integrity was assessed by agarose gel electrophoresis, and concentration was determined by Nanodrop spectrophotometry. Three biological replicates were prepared per group.

Sequencing was performed on the Illumina NovaSeq. 6000 platform by Biomarker Technologies Co., Ltd. Raw reads were subjected to quality control using FastQC to assess per‐base sequence quality, adapter contamination, and GC content. Clean reads were aligned to the reference genome of *A. baumannii* ATCC 19606 (GenBank: AP022836.1) using Bowtie2 with default parameters. Differentially expressed genes (DEGs) were identified using the criteria |log_2_ (fold change) | ≥1 and false discovery rate (FDR) ≤ 0.01. KEGG and GO pathway enrichment analyses of DEGs were performed using the clusterProfiler package in R software.

### Cloning and purification of CRP and CRP^wt^ proteins

The coding sequences of the amplified CRP variant (CRP) and the native CRP protein (CRP^wt^) were cloned into pET28a via Gibson assembly as described previously^51^. The oligonucleotides used in this study are listed in Table S4. The recombinant plasmids, designated pET28a‐CRP and pET28a‐CRP^wt^, were verified by PCR and Sanger sequencing and subsequently transformed into *Escherichia coli* BL21(DE3). An overnight culture was diluted 1:100 into 100 ml of fresh LB medium supplemented with 50 mg/l kanamycin and incubated at 37°C to the mid‐log phase (OD_600_ ~ 0.5). Expression was induced with 0.5 mM isopropyl‐β‐d‐thiogalactopyranoside (IPTG), and cultivation was continued for 18 h at 16°C. His‐tagged recombinant proteins were purified on nickel‐affinity resin (Beyotime) according to the manufacturer's instructions. Purified CRP and CRP^wt^ proteins were analyzed by SDS‐PAGE, and high‐purity fractions were concentrated using 10 kDa MWCO centrifugal filters (Millipore, Sigma‐Aldrich).

### EMSA

The DNA‐binding activities of CRP and CRP^wt^ toward the putative promoter regions of the *paa*, *bas*, and *sfn* clusters were evaluated by EMSA, as described previously^52^. The DNA‐binding activity of CRP toward the putative promoter regions of the *csu*, *mrk*‐like, and *pga* clusters was also examined. Probes (~10 mg/l) were amplified by PCR and purified using a FlaPure Gel Purification Kit (Genesand). The oligonucleotides used in this study are listed in Table S4. EMSA reactions were performed using an EMSA Kit (Beyotime) according to the manufacturer's instructions. Briefly, 10 μl reaction mixtures contained reaction buffer, probe, 6x His‐CRP or 6x His‐CRP^wt^, and 1 nmol cAMP. After incubation at room temperature for 30 min, mixtures were resolved on 6% native polyacrylamide gels containing 1 nmol cAMP. Probes were visualized by staining with GelRed.

### Biofilm assay

Biofilm biomass was assessed using a crystal violet staining assay^53^. Briefly, single colonies of *A. baumannii* ATCC 19606 (pKFAb) and 19606 (pKFAb:*crp‐osmC*) were inoculated into 4 ml of BHI broth and grown overnight at 37°C to the stationary phase. Overnight cultures were adjusted to an OD_600_ of 1.0, and 5 μl of each bacterial suspension was inoculated into wells of a 96‐well polyvinyl chloride plate (Costar) containing 145 μl of BHI supplemented with kanamycin. For each strain and condition, 24 replicate wells were included. Plates were incubated statically at 30°C and 37°C for 24, 48, and 72 h. After incubation, planktonic cells were removed, and the wells were washed three times with distilled water. The plates were then incubated at 37°C for 2 h to fix the biofilms. Biofilms were stained with 220 μl of 0.1% crystal violet for 20 min, after which the stain was removed and the wells were washed five times with distilled water. Plates were dried at 37°C for 1 h, and the bound crystal violet was solubilized with 220 μl of 33% acetic acid. A 200 μl aliquot from each well was transferred to a new 96‐well plate, and biofilm biomass was quantified by measuring absorbance at 590 nm.

For confocal laser‐scanning microscopy, air–liquid interface biofilms, or pellicles, were grown in glass‐bottom chamber slides (Chambered #1.5 German Coverglass System; Nunc). Overnight cultures were adjusted to an OD_600_ of 1.0, and 50 μl of bacterial suspension was inoculated into 900 μl of BHI supplemented with kanamycin. Biofilms were grown statically at 30°C for 24 h. After incubation, biofilms were gently washed with PBS to remove non‐adherent cells and stained in the dark with SYTO9 (5 μM; Invitrogen) and propidium iodide (PI; 30 μM; Mreda) for 15 min. Biofilms were imaged using a Leica TCS SP8 STED confocal laser‐scanning microscope equipped with a 40× objective. SYTO9 was excited at 488 nm, and PI was excited at 561 nm. Confocal image stacks were acquired from representative fields and analyzed using COMSTAT to quantify biofilm architecture^54^.

### Antimicrobial killing assays and sensitivity to disinfectants

Cultures of 19606 (pKFAb), 19606 (pKFAb: *crp‐osmC*), or Δ*crp*Δ*osmC* were grown to a consistent OD_600_ in 4 ml of BHI or BHI with kanamycin for strains with plasmids. Serial dilutions were prepared in PBS, and 5 μl aliquots from each dilution were spot‐plated onto LB agar plates to evaluate bacterial survival. The LB agar was supplemented with a range of antimicrobials and disinfectants at specified final concentrations, which were determined based on preliminary experiments: Meropenem (MEM, 2 mg/l), Ertapenem (ETP, 8 mg/l), Biapenem (BIA, 0.8 mg/l), Aztreonam (ATM, 16 mg/l), Ceftazidime (CAZ, 5 mg/l), Ampicillin (AMP, 800 mg/l), Polymyxin (PMB, 2 mg/l), Ciprofloxacin (CIP, 1 mg/l), Tetracycline (TET, 6 mg/l), Fosfomycin (FOS, 100 mg/l), Chloramphenicol (CAT, 128 mg/l), Benzalkonium chloride (BAK, 0.008%), Hydrogen peroxide (H_2_O_2_, 0.001%), Sodium chloride (NaCl, 4.5%), Cumene hydroperoxide (CHP, 0.3 mM), and Chlorhexidine acetate (CHA, 0.003%), and exposure to UV radiation at 70 J/m^2^.

### Serum killing

Serum was collected from healthy BALB/c mice aged between 8 and 10 weeks. The collected bacteria of 19606 (pKFAb) and 19606 (pKFAb:*crp‐osmC*) were suspended with PBS and adjusted to an OD_600_ of 0.04. 20 μl of culture was mixed with 180 μl of mouse serum. The mixtures were cultured at 200 rpm at 37°C for 3 and 6 h. The samples were serially diluted with PBS, and 10 μl aliquots were spot‐plated onto LB agar plates. The plates were incubated at 37°C and the CFUs were counted the next day. The survival rate was calculated by dividing the CFU at 5 h by the CFU at 0 h. The results were then presented as the fold change of the 19606 (pKFAb: *crp‐osmC*) group compared to the 19606 (pKFAb) group. Experiments were repeated in at least three independent biological replicates. For serum competition assays, the procedure was the same as for *in vitro* competition, except that mouse serum was used as the medium.

### Statistical analysis

Experimental data were analyzed using GraphPad Prism v9 (GraphPad Software, https://www.graphpad.com/). Unless otherwise stated, data are presented as mean ± SD. Multiple‐group comparisons were performed using one‐way analysis of variance (ANOVA), followed by Tukey's multiple‐comparisons test. Competitive index values were compared with the hypothetical value of 1 using two‐tailed one‐sample *t*‐tests. Paired data were analyzed using two‐tailed paired *t*‐tests, whereas independent two‐group comparisons were analyzed using two‐tailed unpaired Student's *t*‐tests. Transcriptomic data were analyzed using standard differential‐expression and functional‐enrichment workflows with correction for multiple testing. Genes with FDR ≤ 0.01 and an absolute fold change ≥2 were considered differentially expressed. *p* < 0.05 was considered statistically significant. Significance is indicated as follows: ns, *p* ≥ 0.05; *p < 0.05; **p < 0.01; ***p < 0.001; and ****p < 0.0001. Sample sizes and replicate types are provided in the corresponding figure legends.

## AUTHOR CONTRIBUTIONS

**Da‐Wei Wei**: Conceptualization; data curation; formal analysis; funding acquisition; investigation; methodology; resources; software; supervision; validation; visualization; writing—original draft. **Jiangqing Huang**: Methodology; software; visualization; writing—review and editing. **Hailing Fang**: Methodology; software; writing—review and editing. **Xinyu Wang**: Methodology; resources; writing—review and editing. **Gang Zhang**: Conceptualization; supervision; writing—review and editing. **Chao Wang**: Supervision; validation; writing—review and editing. **Yuqin Song**: Conceptualization; data curation; formal analysis; methodology; software; visualization; writing—review and editing. **Jie Feng**: Conceptualization; data curation; funding acquisition; project administration; resources; supervision; validation; writing—original draft; writing—review and editing.

## ETHICS STATEMENT

All animal procedures were reviewed and approved by the Ethics Committee of the Institute of Microbiology, Chinese Academy of Sciences (Approval No. APIMCAS2023077). Procedures were conducted in accordance with institutional and national regulations governing the care and use of laboratory animals and followed the ethical principles outlined in the Declaration of Helsinki.

## CONFLICT OF INTERESTS

The authors declare no conflict of interests.

## Supporting information


**Figure S1. A.** Workflow for the analysis of IS‐mediated gene amplification. **B.** Formation of the composite transposon Tn*7691* and subsequent amplification events. **C.** Insertion hotspots associated with Tn*7691*‐mediated amplification.
**Figure S2.** Mapping and analysis of the IS*Aba1* insertion site within Tn*7691*.
**Figure S3.** Body weight changes in mice 24 h after lung infection (A) or intestinal colonization (B). Statistical significance was assessed using a two‐tailed paired t‐test (ns, not significant; ***, *p* < 0.001).
**Figure S4. Pulmonary competition assay in cyclophosphamide‐treated mice. A**, Experimental workflow for cyclophosphamide‐induced immunosuppression and intranasal competition infection with a 1:1 mixture of 19606 (pKFAb:*crp‐osmC*) and 19606 (pKFAb:mCherry) (n = 15 mice). **B**, Body weight changes between 0 and 24 h post‐inoculation. **C**, Competitive index (CI) of 19606 (pKFAb:*crp‐osmC*) versus 19606 (pKFAb:mCherry) in mouse lungs at 24 h post‐inoculation. CI values were normalized to the initial 1:1 input ratio; the dashed line indicates CI = 1. Data are shown as mean ± SD. (n = 15 mice). Statistical significance was determined by a one‐sample *t*‐test against 1; ****, P < 0.0001.
**Figure S5. Expression of IS** ***Aba1*** **‐modified CRP and CRP** ^**wt**^ **proteins and EMSA analysis of their DNA‐binding activity. A**. SDS–PAGE analysis of purified 6His‐CRP and 6His‐CRP^wt^ proteins. P, pellet fraction; S, soluble supernatant fraction; and FT, flow‐through fraction. **B**, EMSA showing that both CRP and CRP^wt^ bind to the putative promoter region of the *bas* cluster. **C, D**, EMSA showing that neither CRP nor CRP^wt^ shows detectable binding to the putative promoter regions of the *paa* cluster (**C**) or the *sfn* cluster (**D**). F, free probe; B, bound probe.
**Figure S6.** EMSA showing no detectable binding of CRP to the putative promoter region of the *mrk*‐like pilus cluster.
**Figure S7. Biofilm biomass of 19606 (pKFAb) and 19606 (pKFAb:** ***crp‐osmC*** **) at later time points.** Biofilm biomass was quantified by crystal violet staining after 48 h and 72 h of static incubation at 30°C or 37°C. Data are shown as mean ± SEM; n = 24 wells per group. Statistical significance was determined by a two‐tailed unpaired Student's *t*‐test; ****, p < 0.0001; ns, not significant.
**Figure S8. Deletion of the** ***crp**‐**osmC*** **gene increases strain resistance to antibiotics.** LB indicates agar without antibiotics, while the following antibiotics were added to other plates: Meropenem (MEM, 2 mg/l), Ampicillin (AMP, 1000 mg/l), Ciprofloxacin (CIP, 1 mg/l), and Chloramphenicol (CAT, 128 mg/l).
**Figure S9. Amplification of the** ***crp**‐**osmC*** **gene does not affect resistance to environmental stresses.** Strains were exposed to UV (70 J/m^2^), H_2_O_2_ (0.001%), NaCl (4.5%), Benzalkonium chloride (BAK, 0.008%), Chlorhexidine acetate (CHA, 0.003%), and Cumene hydroperoxide (CHP, 0.3 mM).
**Figure S10. Putative evolutionary trajectory of Tn** ***7691*** **‐mediated** ***crp**‐**osmC*** **amplification and functional validation in clinical** ***A. baumannii*** **isolates. A.** Numbers and proportions of genomes representing three putative steps in the formation of *crp‐osmC* amplification: IS*Aba1* insertion within *crp*, subsequent IS*Aba1* insertion downstream of *osmC* leading to Tn*7691* formation, and amplification of the Tn*7691*‐flanked *crp‐osmC* region. **B.** Temporal distribution of strains carrying IS*Aba1* insertion in *crp*, Tn*7691*, or *crp‐osmC* amplification according to year of isolation. **C.** Geographic distribution of Tn*7691*‐positive strains and strains with *crp‐osmC* amplification. **D.** Growth curves and *in vitro* competition assays of two clinical ST2 isolates, 21B076 and 21C108, carrying pKF‐ble or pKF‐ble:*crp‐osmC* (n = 5). Competitive indices were compared with the hypothetical value of 1 using a one‐sample *t*‐test; *, p < 0.05, **, p < 0.01. **E**. Spot dilution assays of XH858 carrying pKFAb or pKFAb:*crp‐osmC* on LB agar plates supplemented with meropenem (MEM) or ceftazidime (CAZ).

Table S1. Analysis of IS‐mediated gene amplification in 582 Acinetobacter baumannii complete genomes.
Table S2. Statistical analysis of Tn7691 insertions in the direct repeats (DR) regions.
Table S3. Strains and plasmids in this study.
Table S4. Primer names and sequences used for PCR amplification and qPCR in this study.

## Data Availability

Raw RNA‐seq data have been deposited at Gene Expression Omnibus (GEO) with accession number GSE331122. Custom Python scripts were developed in‐house to mine the complete genomes of 582 *A. baumannii* strains for repeat regions, and are publicly available on GitHub: https://github.com/hailingfang/leaf.
