# Supplementary material for: ISAba1‐mediated crp‐osmC amplification enhances host‐associated fitness in Acinetobacter baumannii
Source: mLife. 2026 Sep 14;5(5):611–25. doi: 10.1002/mlf2.70105 (PMC13574567; doi:10.1002/mlf2.70105)
Supplement: Supplementary file 1 [file MLF2-5-611-s002.docx]

**IS*Aba1*-Mediated *crp-osmC* Gene Cluster Amplification Enhances Nosocomial Fitness in *Acinetobacter baumannii***

Da-Wei Wei^1^, Jiangqing Huang^1, 2^, Hailing Fang^1^, Xinyu wang^1, 2^, Gang Zhang^1^, Chao Wang^1^, Yuqin Song^1, *^& Jie Feng^1, *^

^1^ State Key Laboratory of Microbial Diversity and Innovative Utilization, Institute of Microbiology, Chinese Academy of Sciences, Beijing, China

^2^ College of Life Science, University of Chinese Academy of Sciences, Beijing, China

**Figure S1. A.** Workflow for the analysis of IS-mediated gene amplification. **B.** Formation of the composite transposon Tn*7691* and subsequent amplification events. **C.** Insertion hotspots associated with Tn*7691*-mediated amplification.

**Figure S2.** Mapping and analysis of the IS*Aba1* insertion site within Tn*7691*.

**Figure S3.** Body weight changes of mice 24 hpi after lung infection (A) or intestinal colonization (B). Statistical significance was assessed using a two-tailed paired t-test (ns, not significant; ***, *p* < 0.001).

**Figure S4. Pulmonary competition assay in cyclophosphamide-treated mice. A**, Experimental workflow for cyclophosphamide-induced immunosuppression and intranasal competition infection with a 1:1 mixture of 19606 (pKFAb:*crp-osmC*) and 19606 (pKFAb:mCherry) (n = 15 mice). **B**, Body weight changes between 0 and 24 hpi post-inoculation. **C**, Competitive index (CI) of 19606 (pKFAb:*crp-osmC*) versus 19606 (pKFAb:mCherry) in mouse lungs at 24 hpi post-inoculation. CI values were normalized to the initial 1:1 input ratio; the dashed line indicates CI = 1. Data are shown as mean ± SD. (n = 15 mice). Statistical significance was determined by one-sample *t*-test against 1; ****, P < 0.0001.

**Figure S5. Expression of IS*Aba1*-modified CRP and CRP^wt^ proteins and EMSA analysis of their DNA-binding activity. A**. SDS–PAGE analysis of purified 6His-CRP and 6His-CRP^wt^ proteins. P, pellet fraction; S, soluble supernatant fraction; FT, flow-through fraction. **B**, EMSA showing that both CRP and CRP^wt^ bind to the putative promoter region of the *bas* cluster. **C, D**, EMSA showing that neither CRP nor CRP^wt^ shows detectable binding to the putative promoter regions of the *paa* cluster (**C**) or the *sfn* cluster (**D**). F, free probe; B, bound probe.

**Figure S6.** EMSA showing no detectable binding of CRP to the putative promoter region of the *mrk*-like pilus cluster.

**Figure S7. Biofilm biomass of 19606 (pKFAb) and 19606 (pKFAb:*crp-osmC*) at later time points.** Biofilm biomass was quantified by crystal violet staining after 48 h and 72 h of static incubation at 30°C or 37°C. Data are shown as mean ± SEM; n = 24 wells per group. Statistical significance was determined by two-tailed unpaired Student’s *t*-test; ****, p < 0.0001; ns, not significant.

**Figure S8. Deletion of the *crp-osmC* gene increases strain resistance to antibiotics.** LB indicates agar without antibiotics, while the following antibiotics were added to other plates: Meropenem (MEM, 2 mg/L), Ampicillin (AMP, 1000 mg/L), Ciprofloxacin (CIP, 1 mg/L), and Chloramphenicol (CAT, 128 mg/L).

**Figure S9. Amplification of the *crp-osmC* gene does not affect resistance to environmental stresses.** Strains were exposed to UV (70 J/m²), H₂O₂ (0.001%), NaCl (4.5%), Benzalkonium chloride (BAK, 0.008%), Chlorhexidine acetate (CHA, 0.003%), and Cumene hydroperoxide (CHP, 0.3 mM).

**Figure S10. Putative evolutionary trajectory of Tn*7691*-mediated *crp-osmC* amplification and functional validation in clinical *A. baumannii* isolates. A.** Numbers and proportions of genomes representing three putative steps in the formation of *crp-osmC* amplification: IS*Aba1* insertion within *crp*, subsequent IS*Aba1* insertion downstream of *osmC* leading to Tn*7691* formation, and amplification of the Tn*7691*-flanked *crp-osmC* region. **B.** Temporal distribution of strains carrying IS*Aba1* insertion in *crp*, Tn*7691*, or *crp-osmC* amplification according to year of isolation. **C.** Geographic distribution of Tn*7691*-positive strains and strains with *crp-osmC* amplification. **D.** Growth curves and *in vitro* competition assays of two clinical ST2 isolates, 21B076 and 21C108, carrying pKF-ble or pKF-ble:*crp-osmC* (n = 5). Competitive indices were compared with the hypothetical value of 1 using a one-sample *t*-test; *, p < 0.05, **, p < 0.01. **E**. Spot dilution assays of XH858 carrying pKFAb or pKFAb:*crp-osmC* on LB agar plates supplemented with meropenem (MEM) or ceftazidime (CAZ).
